# Numerical Study of Air Flow Induced by Shock Impact on an Array of Perforated Plates

**DOI:** 10.3390/e23081051

**Published:** 2021-08-16

**Authors:** Lite Zhang, Zilong Feng, Mengyu Sun, Haozhe Jin, Honghui Shi

**Affiliations:** School of Mechanical Engineering and Automation, Zhejiang Sci-Tech University, Hangzhou 310018, China; Z00L99F23@gmail.com (Z.F.); morgenpaloma98@gmail.com (M.S.); haozhejin@zstu.edu.cn (H.J.); hhshi@zstu.edu.cn (H.S.)

**Keywords:** attenuation, numerical simulation, perforated plate, propagation behavior, shock wave, ring vortex

## Abstract

This study is focused on the propagation behavior and attenuation characteristics of a planar incident shock wave when propagating through an array of perforated plates. Based on a density-based coupled explicit algorithm, combined with a third-order MUSCL scheme and the Roe averaged flux difference splitting method, the Navier–Stokes equations and the realizable *k*-ε turbulence model equations describing the air flow are numerically solved. The evolution of the dynamic wave and ring vortex systems is effectively captured and analyzed. The influence of incident shock Mach number, perforated-plate porosity, and plate number on the propagation and attenuation of the shock wave was studied by using pressure- and entropy-based attenuation rates. The results indicate that the reflection, diffraction, transmission, and interference behaviors of the leading shock wave and the superimposed effects due to the trailing secondary shock wave are the main reasons that cause the intensity of the leading shock wave to experience a complex process consisting of attenuation, local enhancement, attenuation, enhancement, and attenuation. The reflected shock interactions with transmitted shock induced ring vortices and jets lead to the deformation and local intensification of the shock wave. The formation of nearly steady jets following the array of perforated plates is attributed to the generation of an oscillation chamber for the inside dynamic wave system between two perforated plates. The vorticity diffusion, merging and splitting of vortex cores dissipate the wave energy. Furthermore, the leading transmitted shock wave attenuates more significantly whereas the reflected shock wave from the first plate of the array attenuates less significantly as the shock Mach number increases. The increase in the porosity weakens the suppression effects on the leading shock wave while increases the attenuation rate of the reflected shock wave. The first perforated plate in the array plays a major role in the attenuation of the shock wave.

## 1. Introduction

When a shock wave propagates through a porous barrier, it is accompanied by a series of complex shock wave propagation and interaction behaviors. A portion of the available energy is converted into internal energy so that the shock wave intensity and thus the impact loading on the downstream components are reduced. In practical engineering applications, porous barriers have significant potential in mitigating the damage caused by shock waves [1,2,3,4,5]. The shock-induced damage or influence happens in many situations such as the influence of explosion waves on the stability of corridor structures in mine tunnels or oil wells [6,7], the damage of shock waves in a high-speed train tunnel to ancillary facilities [8,9], the sonic boom problem caused by supersonic aircraft breaking through the sound barrier [10,11], etc. Therefore, it is of great significance to study the propagation behavior of a shock wave when it passes through a perforated-plate array and reveal the mechanisms that lead to the change in the shock wave intensity.

Britan et al. [12] studied the influence of the geometrical parameters of porous plate on shock wave attenuation, and proposed a one-dimensional inviscid flow model able to provide predictions in good agreement with the experimental results. Yang et al. [13] experimentally studied the dependence of the reflected intensity change due to shock wave impact on a foam barrier on physical and geometric parameters. The experimental results demonstrated that the velocity and stability of the reflected shock wave increased with the increase in the density and length of the foam barrier. Szumowski et al. [14] experimentally and numerically investigated the interaction of a reflected shock wave and a ring vortex occurring in the impingement of a jet starting from a shock tube on a perpendicular solid wall. The main findings included: (1) a toroidal sound wave generates, meanwhile, the shock wave deforms during the interaction; and (2) the impingement of the ring vortex on the wall also causes the generation of sound waves. Kontis et al. [15] conducted a similar experimental study to examine the interaction of shock wave induced vortices with a flat plate and a perforated plate. Apart from the consistent observations with those obtained by Szumowski et al. [14], they observed and analyzed the production of a wall vortex (or secondary vortex ring) for the flat plate case, furthermore, the transmission and reflection of the shock wave respectively flowed by compression waves, and the dissipation of the jet accompanied by the vortices inside and outside the pores for the perforated plate case. Igra et al. [16] used a two-dimensional, compressible, inviscid flow model to numerically study the influence of a series of flat barriers on shock wave attenuation in the channel. It was found that the barriers with different geometric parameters can significantly attenuate the shock waves, especially strong shock waves. Lv et al. [17,18] experimentally studied the attenuation rate of a shock wave impacting on a sand layer and simulated the interaction between the shock wave and the sand by using the Eulerian–Lagrangian coupling method in an explicit finite element solver Autodyn3D. Berger et al. [19] numerically investigated the interaction of a shock wave in a corridor structure with different perforated plates by applying the MSC.Dytran solver in combination with the first- and second-order total variation diminishing (TVD) schemes. The results showed that the shock wave attenuation strongly relied on the porosity and the angle of inclination of the perforated plates. Ram et al. [20,21,22] conducted experiments on shock interaction with an array of perforated plates at different shock Mach numbers, numbers, and geometries of the perforated plates and stand-off distance, and analyzed the dynamic pressure measured on the end wall. He also developed a constitutive model for predicting the same pressure based on an isentropic flow.

This shows that the subject of shock interaction with a porous barrier has been explored extensively. However, most of these investigations focused on the parametric influence of shock wave attenuation. In the process of a shock wave traveling through a perforated-plate array, the propagation and interference of the complex wave system should prominently affect the local change and global attenuation of the shock wave intensity. At present, few studies have carried out systematic and in-depth analysis on the related mechanisms and laws. Aiming at the propagation behavior and attenuation characteristics of a planar incident shock wave when propagating through an array of perforated plates, the flow and geometric models and the numerically computational methods were constructed to simulate the propagation of the shock wave in the core area of the flow field and the evolution of the induced wave and ring vortex systems. The related flow phenomena and mechanisms of local variation of shock wave intensity were analyzed, and the influence laws of shock Mach number, porosity, and plate number on shock wave attenuation are revealed in this study.

## 2. Governing Equations and Numerical Methods

### 2.1. Governing Equations

Three-dimensional time-averaged Navier–Stokes equations were used to describe the high-speed, compressible, viscous, and unsteady air flow field. The governing continuity, momentum, and energy equations are expressed as follows:

(1) ∂ρ/∂t+∇·(ρV)=0

(2) ∂(ρV)/∂t+∇·(ρVV)=−∇p+∇τ

(3) ∂(ρE)/∂t+∇·(ρEV)=−∇(pV)+∇·(κ∇T+Vτ)

where *ρ* is the density; *t* is the time; ***V*** is the velocity vector; ***τ*** is the viscous stress tensor; *E* is the total specific energy (i.e., the sum of specific internal energy and specific kinetic energy); and *k* is the heat conductivity.

The realizable *k*-*ε* turbulence model [23] was adopted in this study because of its high reliability and accuracy, and short computational time relative to other most popular turbulence models including the standard *k*-*ε* model, RNG *k*-*ε* model, SST *k*-*w* model, and RSM in simulations of complex flow fields such as round jets and multiple jets [24], and transient, compressible, and viscous turbulent flows [25]. The turbulent kinetic energy and the rate of turbulent dissipation transport equations are respectively expressed as follows:

(4) $$\frac{\partial }{\partial t}(\rho k)+\frac{\partial }{\partial x_{j}}(\rho ku_{j})=\frac{\partial }{\partial x_{j}}[(\mu +\frac{\mu_{t}}{\sigma_{k}})\frac{\partial k}{\partial x_{j}}]+G_{k}+G_{b}-\rho \varepsilon -Y_{M}+S_{k}$$

(5) $$\frac{\partial }{\partial t}(\rho \varepsilon )+\frac{\partial }{\partial x_{j}}(\rho \varepsilon u_{j})=\frac{\partial }{\partial x_{j}}[(\mu +\frac{\mu_{t}}{\sigma_{\varepsilon }})\frac{\partial \varepsilon }{\partial x_{j}}]+\rho (C_{1}S\varepsilon -C_{2}\frac{\varepsilon^{2}}{k+\sqrt{v\varepsilon }})+C_{1\varepsilon }\frac{\varepsilon }{k}C_{3\varepsilon }G_{b}+S_{\varepsilon }$$

where $C_{1}=\max[0.43,\frac{\eta }{\eta +5}],\eta =S\frac{k}{\varepsilon },S=\sqrt{2S_{ij}S_{ij}}$. In these equations, *x_j_* is the space coordinates; *u_j_* is the gas velocity component; *μ* and *μ_t_* are the molecular and turbulent viscosities, respectively; *σ_k_* and *σ_ε_* are the turbulent Prandtl numbers of the *k* and *ε* equations, respectively; *G_k_* and *G_b_* are the turbulent kinetic energies due to the average velocity gradient and buoyancy, respectively; *Y_M_* is the contribution of pulsating expansion to the total dissipation rate in the compressible turbulent flow; *S_k_* and *S_b_* are the user-defined source terms; *C*_2_ and *C*_1*ε*_ are the model constants, equal to 1.9 and 1.44, respectively.

Finally, the ideal gas state equation was used for the closure of the flow equations. The molecular viscosity of air is calculated with the three-coefficient Sutherland formula.

### 2.2. Geometric Models and Boundary Conditions

**Figure 1:**
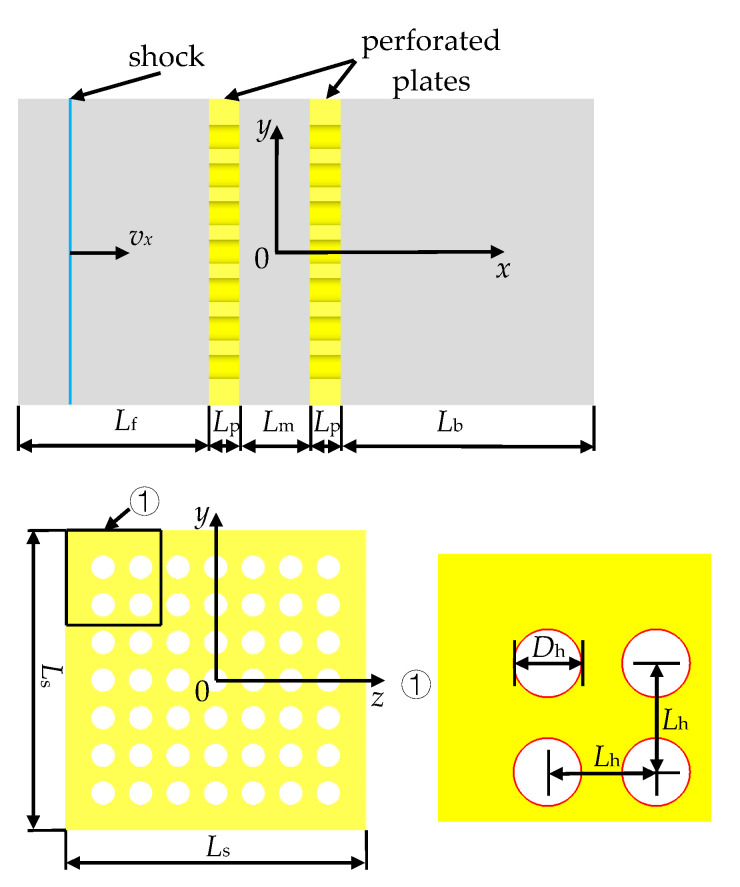
Geometric model.

The geometric model is derived from the internal-channel and perforated-plate structure in Ram’s experimental test section [26], as shown in Figure 1. The thickness of the perforated plate was set as *L*_p_ = 3 mm, the plate spacing *L*_m_ = 7 mm, and the side length of the flow channel was *L*_s_ = 32 mm. In order to avoid the influence of the reflected wave from the inlet and ensure the full development of the leading shock wave at the downstream of the perforated-plate array, the distances from the inlet to the upstream side of the array and that from the end-wall to the downstream side of the array are given as *L*_f_ = 50 mm and *L*_b_ = 70 mm, respectively. The geometric parameters of three types of models marked A, B, and C can be seen in Table 1, in which *L*_h_ and *D*_h_ denote the spacing and diameter of holes while porosity *α* is defined by the ratio of the overall cross-sectional area of the holes to that of the flow channel.

**Figure 2:**
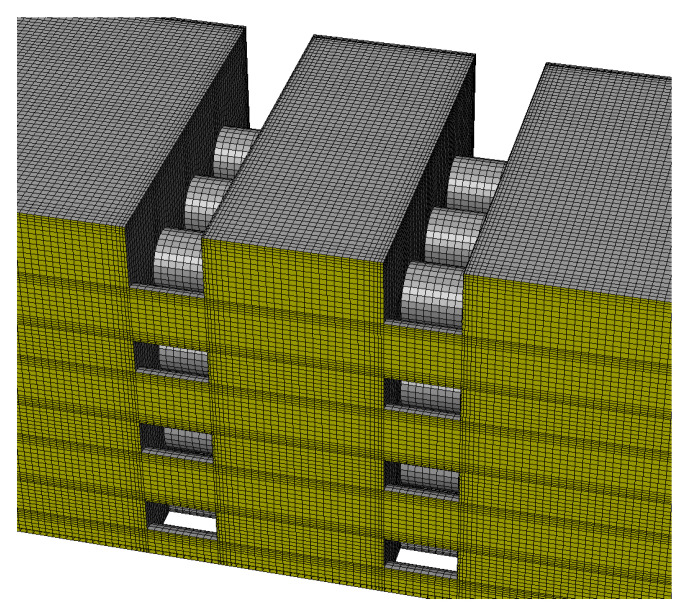
Grids in the computational domain.

The origin of the Cartesian coordinates corresponds to the center of the cross section at 3.5-mm downstream of the first perforated plate. A planar incident shock wave propagates along the positive direction of the *x*-axis. A quarter of the whole is selected as the computational domain due to the symmetry of structure of the flow channel, and meanwhile, structured hexahedral grids are used for the discretization of the computational domain to reduce the computational cost and improve the grid quality. The grids in the core area of the flow channel are illustrated in Figure 2. According to the requirement for a *k*-*ε* turbulence model that the first layer grids should fall in the logarithmic region of a turbulent boundary layer (in general, y^+^ = 30–300), the minimum grid sizes suitable for all the present computational conditions can be estimated as around 0.02–0.2 mm by using a characteristic length *L*_f_ or *L*_b_, post-shock flow and thermodynamic parameters of the incident shock wave, and the dynamic viscosity of air. In actuality, the only minimum grid size Δ*r*_min_ in directions perpendicular to the walls is set as 0.1 mm, while the size of global grids (non-boundary layer grids) is selected as 0.2, 0.3, or 0.4 mm corresponding respectively to a total cell number of 6.02, 2.03, or 0.78 million for verification of grid independence. The boundary layer grids are smoothly transitioned to the global grids by regulating an amplification factor below 1.2. The grid quality indexes of above 0.7 are achieved totally, which facilitates the stability and convergence of the numerical computations.

**Figure 3:**
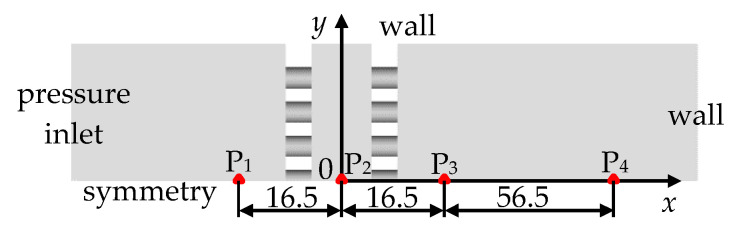
Setting of boundary conditions and layout of monitoring points.

Figure 3 shows the boundary conditional setting and the layout of monitoring points of dynamic pressure. Pressure inlet and symmetrical boundary conditions were used for the inlet and symmetrical planes, respectively. A no-slip, adiabatic wall condition was applied to the remaining boundaries. The monitoring points P_1_ and P_3_ were located at 10-mm upstream and downstream of the array of perforated plates, respectively. The position of P_2_ was located at the origin of the coordinates. The layout of the monitoring point P_4_ was mainly based on the following two considerations. First, the position of P_4_ should not be too close to the array to avoid a downstream region where the flow parameters change drastically. Second, we should prevent the shock wave from traveling too long a distance, causing an additional attenuation of the shock wave due to the viscous effects of the fluid. The layout is determined through test calculations and analysis. The monitoring point P_4_ was situated in a position at 50.0-mm downstream of the array. This was employed to quantitatively evaluate the attenuation of the leading shock wave after passing through the perforated-plate array.

### 2.3. Numerical Methodology

The governing equations were discretized with a finite volume method, and numerically solved with a density-based, coupled, explicit solver. In order to capture the traveling waves and ring vortices more accurately, a third-order MUSCL scheme was applied in the discretization of the continuity, momentum, and energy equations, while the Roe averaged flux difference splitting method was used for the convective flux.

The initialization of the flow field was achieved through a “PATCH” instruction. Specifically, an initial condition of a traveling shock wave can be set by dividing the computational domain into high- and low-pressure two subdomains in which the flow parameters have respectively different uniform distributions. The initial parameters on both sides of the shock front are calculated by the shock tube principle. Table 2 shows the parameters in the initial flow fields studied in this work, where *M*_S_ denotes the incident shock Mach number, subscript “2” is the post-shock parameter(s), and the subscript “0” is the post-shock stagnation parameter(s). Furthermore, the initial pressure and temperature in the low-pressure area were set as *p*_1_ = 101,325 Pa and *T*_1_ = 300 K, respectively. The Courant–Friedrichs–Lewy stability criterion for an explicit solver requires that the adopted Courant number (denoted by *CFL*) should not be greater than unity. In the present simulations, the appropriate range of time step can be determined by

(6) $$\Delta t\leq CFL\frac{\Delta r_{\min}}{v_{2}+c_{2}}$$

where *c*_2_ is the post-shock sound speed. We obtained Δ*t* ≤ 1.4 × 10^−7^ s. In order to balance the computational convergence and economy, the Courant number was set as *CFL* = 0.8, and thus the time step Δ*t* = 2.5 × 10^−^^8^ s through test calculations.

## 3. Validation of Numerical Methods

**Figure 4:**
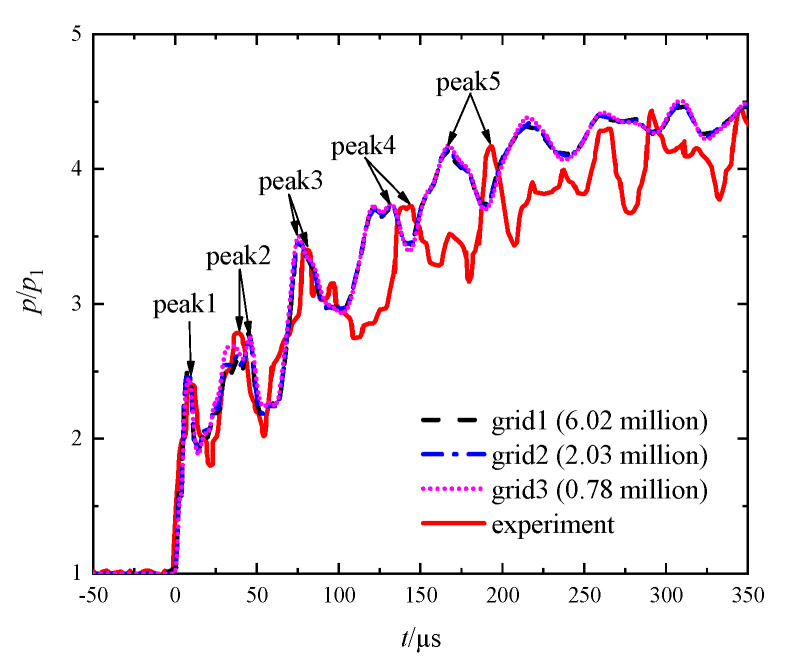
Comparison of the instantaneous pressures from the numerical calculations on different grids and from Ram’s experiment [26].

A comparison of the transient pressures at the center of the end wall from the numerical calculations on different grids and from Ram’s experiment [26] is shown in Figure 4, in which the time zero was set as the arrival time of the incident shock wave. It was found that the computational results obtained on the three sets of grids showed little difference. We believe that the grid independence was achieved on each set of grids denoted by grid1 to grid3 in Figure 4. In order to balance the resolution of the structure of the wave system and the computational cost, grid2 was selected for the subsequent calculations.

On the other hand, the variation trend of each computational instantaneous pressure was very consistent with that of Ram’s experimental [26] one. Table 3 lists the first five peak values both from the computation on grid2 and from Ram’s experiment [26], and the corresponding relative errors. It was found that the maximum relative error was only 3.33%. Furthermore, it was observed from Figure 4 that the calculated peak times starting from peak2 were smaller than the corresponding experimental data. In the present simulation, we treated each of the perforated plates as a solid wall, meaning that shock or compression waves propagated through it with an infinite speed. However, the real material of the plates (Perspex) limits the propagating speed of such waves within it. As a result, the peak times from the simulation appeared to be earlier than that in the real situation, and the difference gradually enlarged with time. However, the instantaneous pressures from both the simulation and the experiment asymptotically tended to the same ultimate value. Furthermore, as described above, the difference between the peak values of the instantaneous pressures was very small, which demonstrates the validity of the numerical methods.

## 4. Results and Analysis

### 4.1. Flow Phenomena

**Figure 5:**
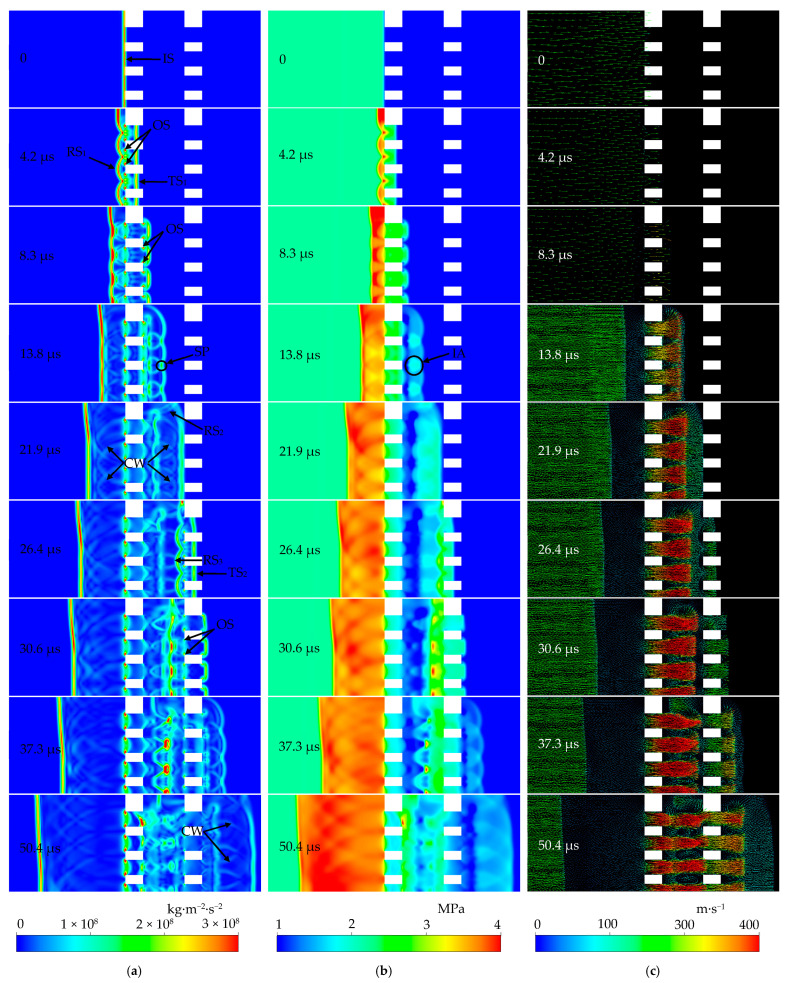
(**a**) Instantaneous pressure gradient; (**b**) Instantaneous pressure; (**c**) Velocity vector.

The development of the air flow field induced by shock impingement on a perforated-plate array is a highly unsteady process. Figure 5a–c shows the calculated instantaneous pressure gradient, pressure, and velocity vector distributions under the condition of the model B perforated-plate array and *M*_s_ = 1.41, respectively.

It can be seen that at the *t* = 0 moment, the planar incident shock front (abbreviated as IS) arrived exactly at the frontal edge of the array and manifested as a peak pressure gradient or a strong pressure discontinuity. The distributions of pressure and velocity vector on both sides of the IS were still uniform. At the *t* = 4.2 μs moment, a series of bow-shaped reflected shock waves RS_1_ propagating upstream and transmitted shock waves TS_1_ propagating downstream in the holes formed after the IS impacted the first perforated plate. The post-shock pressure of the TS_1_ changed slightly relative to that of the IS, meaning that the change of the shock wave intensity was insignificant. In addition, nearly stationary oblique shock waves appeared at the upstream edges of the inlet of each hole in the first plate, surviving the whole of the studied time.

At the *t* = 8.3 μs moment, the transmitted shock wave TS_1_ completely passed through the hole channels and entered the area between the two plates. Due to the sudden expansion structure of the flow channel at the rear side of the first plate, the TS_1_, which is subject to disturbances from the outlet edges of the holes, diffracted so that its shock front shifted from the original planar structure to a curved surface consisting of a series of “mushroom head” shaped substructures, which are shown as bow-shaped shock waves in Figure 5a,b. A jet starting from each hole is preceded by each of the bow shock waves. Consequently, the pressure inside the holes decreases with the discharge of the jets. Oblique shock waves begin to appear at the downstream edges of the outlet of each plate. According to the study of Baird [27], the generation of these oblique shocks is due to the acceleration of expansion waves inside the hole. Moreover, because of shock–shock and shock–wall interactions, the fluctuant shock front of the RS_1_ continuously levels out to develop into a nearly planar structure. The portion close to a side wall propagates faster than that close to the symmetric plane. As a result, the RS_1_ shock front gradually becomes an inclined plane. The main reason is that the shock reflection from a near-wall, corner region is stronger than that from the remaining non-passage, solid body regions at the upstream side of the perforated plate. This is well proven by the significant, locally high pressure in the post-shock region at both the *t* = 4.2 μs and *t* = 8.3 μs moments.

At the *t* = 13.8 μs moment, the first significant decrease in the intensity happens to the TS_1_, as demonstrated by the continuous decrease in the post-shock pressure. As the transmitted bow-shaped shock waves extend, the interference happens first to the neighboring ones. Consequently, locally high pressures appear in the interference areas IA (i.e., the post-shock), overlapping portions of the TS_1_. Meanwhile, a “strengthening point” SP appeared at a position where two neighboring transmitted bow-shaped shock fronts intersect. The superimposed effects cause increases in the local shock wave intensity and propagation speed, meaning that the SP moves downstream faster than the remaining portions of the TS_1_ front. This explains the similar phenomena that the fluctuant fronts of the TS_1_ and RS_1_ become smooth gradually. Each jet gradually develops to form a nearly toroidal, locally high-speed region. A vortex ring is generated due to the development of a shear layer between the jet and the external fluid. The neighboring portions with opposite vorticities of two ring vortices (i.e., vortex cores) merge into a “heart” shaped structure, as shown in Figure 5c. This merging of vortex cores dissipates the energy of themselves and the surrounding oblique shock and compression waves.

Furthermore, due to the suppression effects to the flow from the first perforated plate, the pressure upstream of the first plate maintains a level higher than that of the initial post-shock pressure of the IS. This provides conditions for the further development of the jets.

At the *t* = 21.9 μs moment, the transmitted shock wave TS_1_ only reached the upstream side of the second perforated plate. The transmitted shock wave from the hole in the first perforated plate closest to the wall encounters the wall, thus leading to the formation of a reflected shock wave RS_2_, which interferes with the TS_1_. As a result, the local pressure near the wall increases. At this point, the pressure inside the holes of the first plate has basically decreased to the level equivalent to the initial post-shock pressure of the IS. Reflected and transmitted compression waves following the RS_1_ and the TS_1_ can be clearly seen upstream and downstream the first plate, respectively. This observation agrees with the experimental work of Kontis et al. [15]. Citing the work of Torrens and Wrobel [28], it is believed that the compression waves are generated as a result of the reflections of the transmitted shock (i.e., TS_1_ here) in both directions inside the perforated medium. Furthermore, the reflected and transmitted shocks are strengthened due to the superposition of these compression waves. Furthermore, the high-speed region of each jet becomes simply connected and develops further, dragging the vortex cores travelling downstream. The streamwise size of each merged vortex core structure is stretched. Meanwhile, the diffusion of vorticity dissipates the energy of each ring vortex and the surrounding waves.

At the *t* = 26.4 μs moment, a transmitted shock wave TS_2_ propagating downstream through the holes in the second perforated plate and a bow-shaped reflected shock wave RS_3_ comes into being simultaneously. Similar, but weaker oblique shocks appeared at the upstream edges of the inlet of each hole in the second plate.

In the time range of 30.6 ≤ *t* ≤ 37.3 μs, each jet flow that discharges from the first perforated plate develops an oblique shock pattern. The RS_3_ first collides with the ring vortices. As a result, multiple layers of shock waves are generated. This observation is also reported in the experimental study of Kontis et al. [15]. Furthermore, the local intensity of shock wave at intersections of the downstream cross section of the vortex cores with the central lines of the holes is remarkably increased. This finding is consistent with that from the numerical simulations by Takayama et al. [29]. They attributed the local increase in shock intensity to a double-step mechanism. The head-on collision of the reflected shock with the high-speed flow inside the vortex causes a first slight intensification. Then, a second intensification is due to the convergence of the outside diffracted wave around the vortex on the intensified portion near each central line. Thereafter, the RS_3_ with the multiple layer structure continues to propagate upstream and interacts with the oblique shock pattern within each jet flow, producing “α”-shaped shock waves. The oblique shocks represented by the two legs of “α” are strengthened during this interaction. The evolution of the transmitted shock wave TS_2_ is basically the same as that of the TS_1_. Successive interference of the TS_2_ from different holes changes the post-shock parameters of the flow field. It is inferred from the pressure gradient and pressure contours (Figure 5a,b) that the TS_2_ significantly attenuated relative to the IS. In other words, the second remarkable decrease happened to the intensity of the leading shock wave.

At the *t* = 50.4 μs moment, one part of the reflected shock wave RS_3_ collides with the oblique shocks at the hole outlets of the first perforated plate. Consequently, the local shock intensity of this part is increased and propagates upstream through the holes. The remaining part is reflected by the plate. As a result, the shock intensity of the reflected part decreases due to the viscous dissipation and the energy loss during the collision process. The angle between the transmitted shock TS_2_ and the wall reaches the critical value for transition from regular reflection to Mach reflection. As a result, a Mach reflection starts to form. Each jet originating from the first perforated plate is stretched along the travelling direction. The inside flow velocity decreases due to dissipation effects as it moves downstream further. The merged vortex core structure splits into two individual ones. This irreversible process naturally brings some amount of energy dissipation. It is found that the size of each vortex core becomes greater than that at previous moments because of the diffusion of vorticity. This is another dissipation mechanism of the ring vortices. It should be noted that the interaction of the neighboring vortex cores should also contribute significantly to the dissipation of the ring vortices. The separated vortex cores lie side by side in the transverse direction, except for the farthest two from the symmetric centerline. We believe that the asymmetry due to the sidewall leads to the change in their arrangement pattern. A weaker behavior of ring vortices and jets induced by the TS_2_ similar to that described for the moment of *t* = 13.8 μs occurs downstream of the second plate.

In addition, it is found that the upstream pressure of the first perforated plate maintains a high level for an extended period of time. Although the upstream pressure is also locally increased near the second plate due to the reflection of the TS_2_, both the pressure level and the duration were significantly below that corresponding to the first plate, indicating that the first perforated plate plays a major role in the inhibition of the flow.

**Figure 6:**
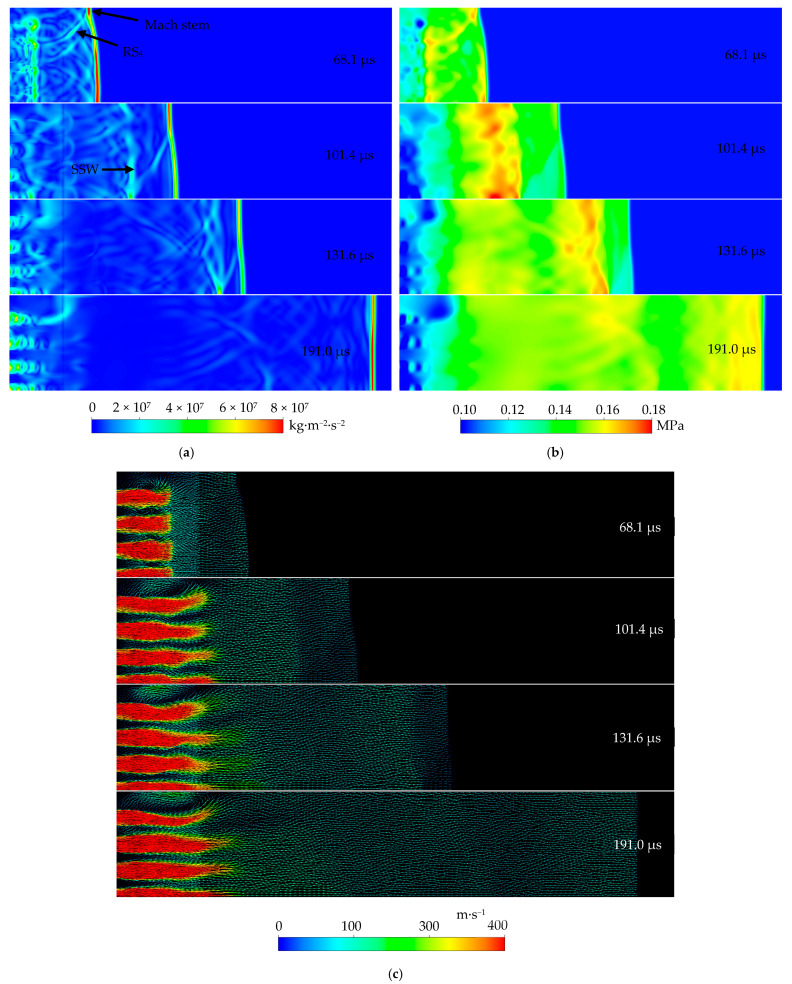
(**a**) Instantaneous pressure gradient; (**b**) Instantaneous pressure; (**c**) Velocity vector.

Figure 6a–c presents the later instantaneous pressure gradient, pressure, and velocity vector distributions at the downstream of the perforated-plate array, respectively. It can be found that in the time range of 68.1 ≤ *t* ≤ 101.4 μs, Mach reflections of the transmitted shock waves from the different holes in the second plate occur at the side wall, characterized by a Mach stem perpendicular to the wall, a curved reflected shock wave RS_4_, and the remaining TS_2_. Surprisingly, the TS_2_ is followed by secondary shock waves SSW. We infer that these SSW are formed due to two mechanisms. One is the superimposed effects of multiple Mach reflections of the transmitted shock waves from the holes in the second plate except for the one closest to the side wall. The other is the superposition of the transmitted compression waves caused by the reflections of the transmitted shock (i.e., TS_2_ here) inside the holes of the second plate. The latter was proposed in single shock tube studies of Torrens and Wrobel [28] and Kontis et al. [15]. The Mach stem extends toward the symmetric plane, and the SSW chases the leading TS_2_. Consequently, the local intensity of the TS_2_ near the side wall is increased, thus travels faster than the remaining portion, flattening the TS_2_ front. The post-shock pressures of both the RS_4_ and SSW are increased. Because the SSW moves faster than the leading TS_2_, the former catches up and superimposes with the latter at a moment of around 164.1 μs, forming a new, stronger leading shock wave. Additionally, jets discharged from the holes in the second plate continue to develop. The vortex cores induced by the TS_2_ gradually become weak because of the dissipation mechanisms described above.

The long-time interference of the TS_2_ from different holes makes the distribution of the post-shock pressure tend to be uniform. On the other hand, before being affected by the reflected shock wave from the end wall, a nearly steady jet flow with a typical oblique shock pattern develops. Baird [27] found a similar flow pattern and attributed its generation to the acceleration of expansion waves inside the shock tube (i.e., each hole in the second plate). As a result, almost completely quiescent pressure subdomains in a regular arrangement, in which each line alternates between low and high pressures repeatedly following a hole in the second plate, come into being inside each of the jets. It can be noticed that the leading transmitted shock wave propagated downstream, far away from the second plate at the late stages. Therefore, these jets should not be induced by the leading shock directly and different from those generated at earlier stages (e.g., *t* = 50.4 μs). We believe that an oscillation chamber for the inside dynamic wave system is produced between the two perforated plates, making the internal pressure maintain a temporarily stable level much higher than that downstream the second plate at these stages. It is a high enough pressure drop between two sides of the plate that maintains the existence of such jets.

In summary, in the case of shock impact on an array of perforated plates, the reflection, transmission, diffraction, interference behaviors, and the superimposed effects of compression waves and secondary shock waves following the leading transmitted shock wave are the main reasons for the process of attenuation, local enhancement, attenuation, enhancement and attenuation experienced by the leading shock wave. Additionally, many flow phenomena occur in this process such as Mach reflections of the transmitted shock wave, generation of the secondary shock waves, superposition of reflected compression waves and the reflected shock wave, production of jet flows and ring vortices, reflected shock interactions with the ring vortices and oblique shock patterns in the jets, interaction of neighboring vortex cores, deformation and travelling of the ring vortices, occurring of nearly steady jets, and so on. For further understanding regarding the details, the reader is directed to the excellent studies of Kontis et al. [15], Baird [27], Torrens and Wrobel [28], and Takayama et al. [29], among others. In this study, we only focused on the quantitative analysis for the attenuation of the leading shock wave.

### 4.2. Effect of Shock Mach Number

**Figure 7:**
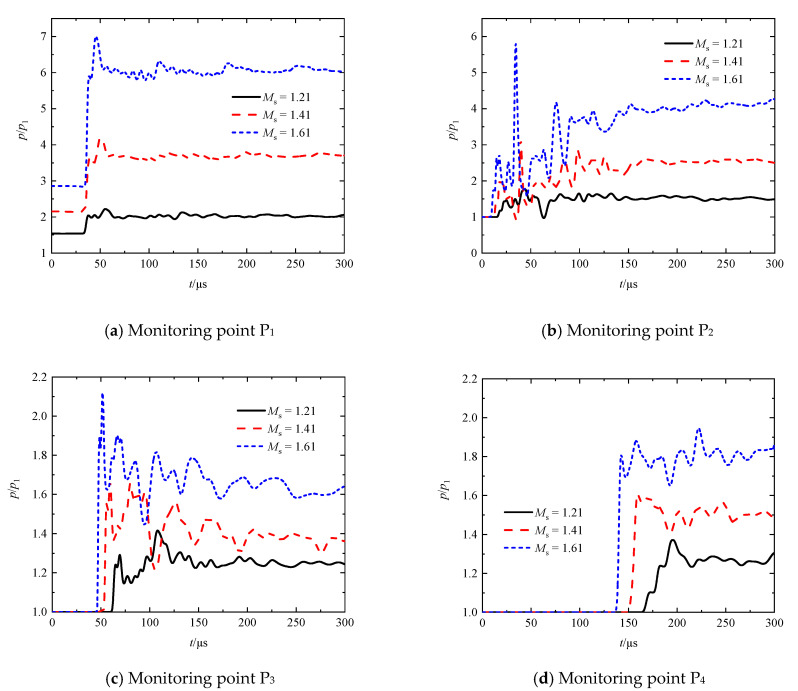
Transient pressures at monitoring points P_1_, P_2_, P_3_, and P_4_ at different shock Mach numbers.

Figure 7a–d presents the transient pressures at different shock Mach numbers (*M*_s_ = 1.21, 1.41, and 1.61) for an array of type B perforated plates at monitoring points P_1_, P_2_, P_3_, and P_4_, respectively. It can be seen in Figure 7a that a pressure spine following a short platform appears on each curve of pressure in the time range of 37.8 ≤ *t* ≤ 55.1 μs. It can be concluded from the peak values of the first spines that the intensity of the reflected shock wave RS_1_ increases with the increase in the incident shock Mach number. In the case of *M*_s_ = 1.61, pressure spines with decreasing amplitudes appear periodically. It can be inferred that the first spine is due to the interference of neighboring reflected shock waves, while the following ones are caused by the reflected compression waves. Furthermore, the stronger the incident shock intensity, the more obvious the spine structures and the more active the interference behavior.

It is observed from Figure 7b that first pressure spines appear on the pressure curves corresponding to the monitoring point P_2_ in the time range of 17.6 ≤ *t* ≤ 24.9 μs. By comparing the peak values of the spines, it can be concluded that the transmitted shock intensity increases with the increase in the incident shock Mach number. Within the following around 20 μs, the transient pressure experiences a process consisting of first a rapid decline, small rise, second decline, and drastic rise. Combining Figure 5a–c, it can be inferred that the first decline and the subsequent rise are caused by the diffraction of the TS_1_ and its interference, respectively. The second decline is due to the arrival of the high-speed and low-pressure region inside the jet at the position of P_2_, while the drastic rise happens when the RS_2_ arrives at the same position. In the time range of 29.4 ≤ *t* ≤ 35.8 μs, the extent of pressure drop for *M*_s_ = 1.41 is more remarkable relative to that for the remaining cases. This is because the collision of the ring vortex and the RS_2_ happens at the downstream region of P_2_, thus the transient pressure reflects the minimum level in the jet in the *M*_s_ = 1.41 case. In the *M*_s_ = 1.21 case, the jet develops relatively slowly, whereas in the *M*_s_ = 1.61 case, both the TS_1_ and the RS_2_ move faster. The similar consequences in the latter two cases conclude that the collision happens at a position close to the vertical split of the two plates, leading to the formation of locally high-pressure regions and therefore the appearance of more distinct second pressure spines in the time range of 34.6 ≤ *t* ≤ 43.2 μs. Thereafter, as the RS_2_ dissipates and degrades gradually, the oscillation of pressure at the P_2_ position is mitigated. Furthermore, the decrease in the incident shock Mach number is beneficial for the flow field to reach a uniform and steady state more rapidly.

It can be seen from Figure 7c that each pressure curve corresponding to monitoring point P_3_ has an obvious spine in the time range of 51.6 ≤ *t* ≤ 69.2 μs, and the peak value decreases significantly compared with the transient post-shock pressure of the IS (see the headmost horizontal straight line in Figure 7a). Moreover, the pressure declines more seriously as the incident shock Mach number increases, meaning that the attenuation of the leading shock wave is more remarkable, and the suppression effects on the shock wave are stronger. Furthermore, the pressure spines occur repeatedly in the studied time range of *t* ≤ 300 μs. We believe that the first spine is due to the interference of neighboring transmitted shock waves, while the remaining are caused by the passage of transmitted compression waves and secondary shock waves following TS_2_. It is also observed from Figure 7d that the pressure amplitude decreases gradually, thus each pressure curve finally maintains at quite a stable level. The decrease in the incident shock Mach number leads to the decrease in the amplitudes of pressure oscillations when approaching a steady state. In combination with the analysis for the generation of the nearly steady jets downstream the array of perforated plates, it can be concluded that these small-amplitude pressure oscillations are mainly caused by the oscillation waves travelling downstream with the fluid medium above-mentioned.

**Figure 8:**
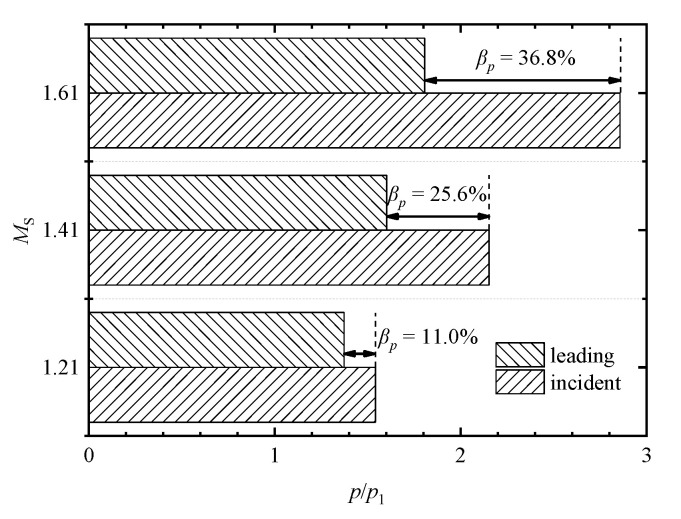
Comparison of pressure-based attenuation rates of the leading shock wave at different incident shock Mach numbers.

Figure 8 shows a comparison of attenuation rates of the leading shock wave at the different incident shock Mach numbers. The pressure-based attenuation rate *β_p_* is defined as

(7) $$\beta_{p}=1-\frac{p_{L}}{p_{2}}$$

where *p*_L_ is the post-shock pressure of leading shock wave at monitoring point P_4_. In the *M*_s_ = 1.21 case, the post-shock pressure of leading shock wave *p*_L_/*p*_1_ is 1.37, thus the shock attenuation rate *β_p_ =* 11.0%. In the *M*_s_ = 1.41 and 1.61 cases, the *p*_L_/*p*_1_ are equal to 1.60 and 1.81, respectively, and therefore the pressure-based attenuation rates are calculated as 25.6% and 36.8%, respectively. This indicates that the increase in the incident shock Mach number improves the attenuation of the leading shock wave during its propagation through an array of perforated plates.

### 4.3. Effect of Porosity

**Figure 9:**
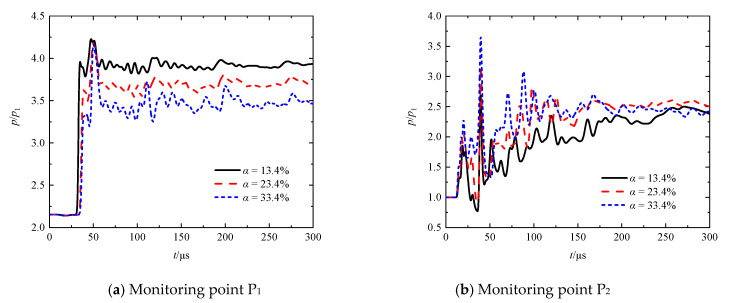

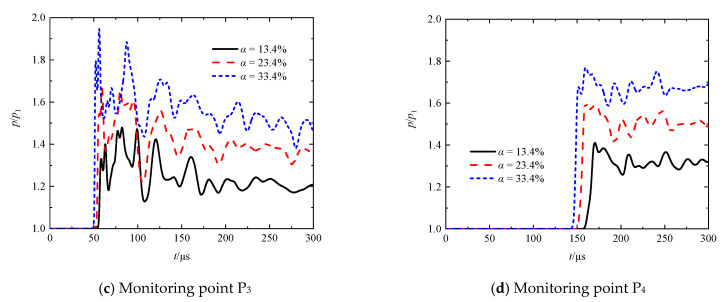
Transient pressures at monitoring points P_1_, P_2_, P_3_, and P_4_ at different porosities.

Figure 9a–d presents the transient pressures at *M*_s_ = 1.41 for different porosities (*α* = 13.4%, 23.4%, and 33.4%) at the monitoring points P_1_, P_2_, P_3_, and P_4_, respectively. It can be observed from Figure 9a that the first pressure spines with small amplitudes appeared in the time range of 47.5 ≤ *t* ≤ 50.4 μs on the curves corresponding to the monitoring point P_1_. Each of these spines is caused by the arrival of a reflected shock wave RS_1_. By comparing the peak values of the spines, it can be found that the intensity of the reflected shock wave increases with decreasing porosity. This can be readily explained as the decrease in the porosity of the perforated plates restrains the flow capacity. The first spine on each curve is followed by a series of spines with gradually decreasing amplitudes. Particularly for the minimum porosity case, a nearly periodic appearance of the pressure spines is evident. A notable phenomenon is that the decrease in the porosity appears to decrease the amplitude of the first pressure spine. We infer that this is related to the shapes of the individually reflected shock waves when they interfere with the neighboring ones.

It can be seen from Figure 9b that the evident first pressure spines appear in the time range of 18.9 ≤ *t* ≤ 20.8 μs on the curves corresponding to the monitoring point P_2_. Each of these spines is due to the arrival of a transmitted shock wave TS_1_. According to the peak values of these spines, it can be inferred that the transmitted shock intensity increases with the increase in porosity. As delineated above, the first spine is followed by the stages including first decline, small rise, second decline, and sharp rise within the subsequent, around 20 μs. It was found that the amplitude of the first pressure decline for the minimum porosity or of the first pressure rise for the maximum porosity is more remarkable than that in the other cases. The pressure spine with the maximum peak value can be seen on each pressure curve in Figure 9b within a time range of 39.4 ≤ *t* ≤ 39.7 μs. Furthermore, the peak value increases with increasing porosity. All of these can be explained by the effect of porosity on the flow capacity summarized above. Furthermore, it can be found that the pressure recovery in the region between the first two plates decreases as the porosity increases. One can see that the transient pressure for the minimum porosity case tends to overtake that for the other cases. The reflected shock wave RS_2_ and its subsequent reflected waves propagate back and forth inside an oscillation chamber between the two plates. The decrease in the porosity is beneficial for the reflection, while adverse to the transmission of these waves. As a result, the superimposed pressurization effect due to the repeated shock reflections is increased as the porosity decreases.

It can be seen from Figure 9c,d that the evident first pressure spines appear in the time ranges of 52.3 ≤ *t* ≤ 59.3 μs and of 151.6 ≤ *t* ≤ 170.2 μs on the curves corresponding to the monitoring point P_3_ and P_4_, respectively. By comparing the peak values of these spines, we can conclude again that the transmitted shock intensity increases as the porosity increases.

**Figure 10:**
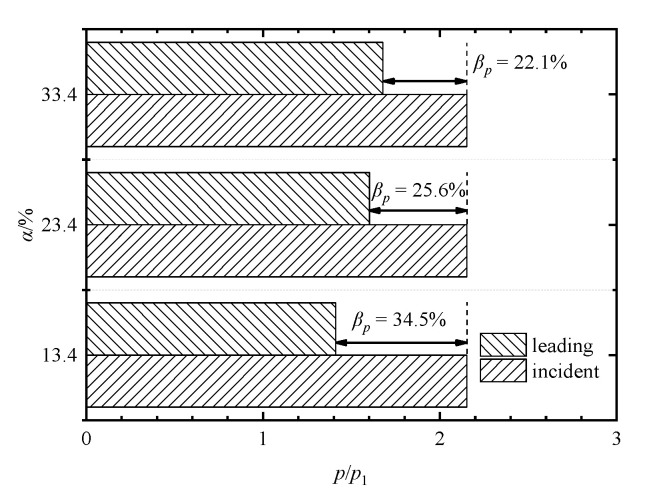
Comparison of pressure-based attenuation rates of the leading shock wave at different porosities.

A comparison of pressure-based attenuation rates of the leading shock wave at different porosities is shown in Figure 10. It can be found that when *α* = 13.4%, 23.4%, and 33.4%, the pressure-based attenuation rates are 34.5%, 25.6%, and 22.1%, respectively. Thus, it can be summarized that the decrease in the porosity strengthens the inhibition effect of the perforated plates on the leading shock wave, and therefore decreases its intensity more significantly.

### 4.4. Effect of Plate Number

**Figure 11:**
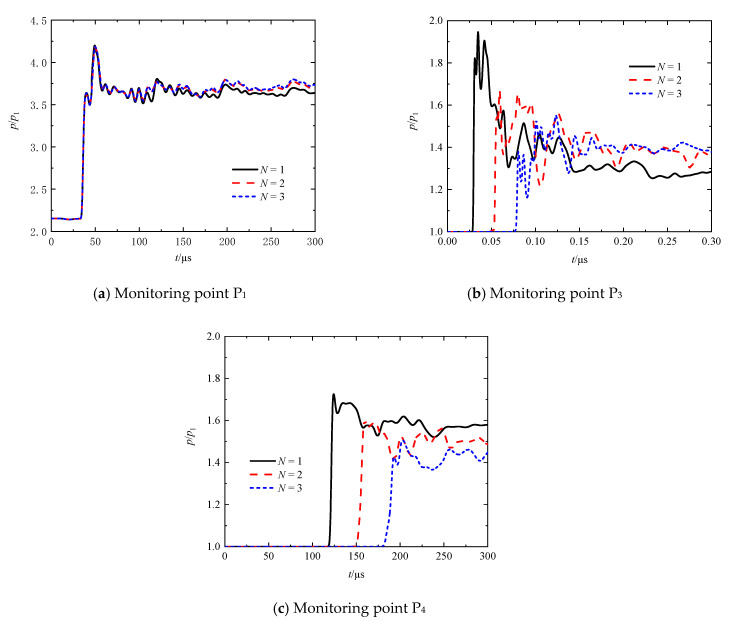
Transient pressures at monitoring points P_1_, P_3_, and P_4_ at different plate numbers.

Figure 11a–c shows the transient pressures for different plate numbers (*N =* 1, 2, and 3) at *M*_s_ = 1.41 at the monitoring points P_1_ and P_3_, respectively. Recall that the monitoring points P_1_, P_3_, and P_4_ are located at 10-mm upstream and downstream of the array, respectively, regardless of what the perforated-plate number is. It can be seen that all the curves completely overlap over the time range before a moment of around 107.6 μs, and the discrepancy of the curves only appeared hereafter. The later discrepancy is due to the influence of the reflected shock waves from the second and third plates (if they exist). The increase in plate number appears to cause more frequent oscillations and higher ultimate values of pressure because the transient pressure is disturbed and increased slightly due to the arrival of the reflected shock (or compression) waves successively.

The first pressure spines appeared at the monitoring point P_3_ for *t* = 34.8, 59.3, and 80.7 μs moments, respectively. By comparing the peak values of the spines, it can be seen that the pressure-based attenuation rates of the leading shock wave increased with the increase in the plate number. For the single- and double-plate cases, the peak values of the second spines were larger than those of the first ones, and the interval times between the first and second spines were significantly shorter than that for the triple-plate case. It can be inferred that the secondary shock waves chase the leading transmitted shock wave more closely and have larger intensities for the single- and double-plate cases than that for the triple case. The secondary shock waves originate from Mach reflections and compression waves induced by the transmitted shock wave. Therefore, the increase in plate number naturally leads to such consequences of secondary shock waves. Significant pressure increase relative to the first spine was observable later in the *N* = 2 and 3 cases. However, this does happen in the *N* = 1 case. One also can find that the increase in plate number increases the ultimate values of pressure. As described above, reflected shock waves are able to propagate back and forth between plates. Consequently, the pressure in such an oscillation chamber between the plates is increased. The influence on the increased pressure will be migrated downstream with the fluid medium or through compression waves. For the studied plate numbers, the increase in plate number improved the pressure recovery ability to maintain a high-pressure level. Additionally, it is found from Figure 11c that the maximum peak values have been shifted from positions of the second spines to those of the first spines in the single- and double-plate cases, however the situation in the tripe-plate case happens exactly in an opposite way. We infer that the superposition of the following secondary shock waves and the leading transmitted shock wave has happened in the former cases rather than in the latter case due to the different intensities of the secondary shock waves.

**Figure 12:**
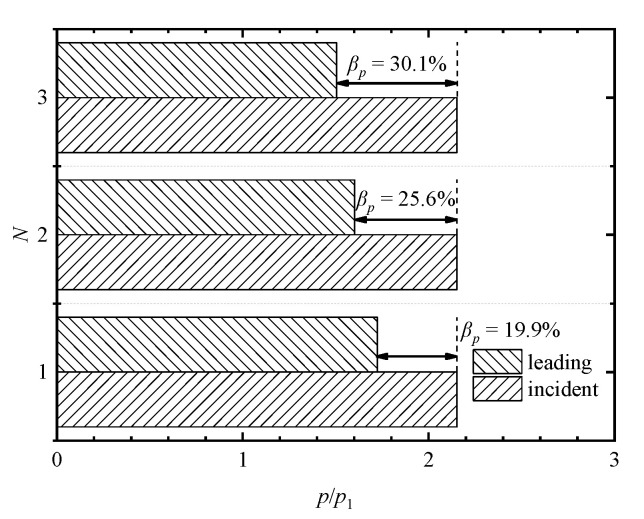
Comparison of pressure-based attenuation rates of the leading shock wave at different plate numbers.

A comparison of pressure-based attenuation rates of the leading shock wave at different plate numbers is shown in Figure 12. The attenuation rates corresponding to the single-, double-, and triple-plate cases are 19.9%, 25.6%, and 30.1%, respectively. It is found that the pressure-based attenuation rate increases with the increase in the plate number. The attenuation of the leading shock wave caused by the subsequent addition of each plate is much more insignificant than that caused by the first plate, indicating that the first plate plays a major role in the shock wave attenuation.

## 5. Entropy Analysis

For the issue of an incident shock interaction with an array of perforated plates, except for the complex propagation behavior of the shock wave, many irreversible phenomena are involved such as the afore-described interaction of the reflected shock from a perforated plate with ring vortices or with oblique shock waves, production of jet flows with oblique shock patterns, merging and splitting of vortex cores, deformation of ring vortices due to vorticity diffusion, generation of secondary shock waves, superposition of compression waves with the preceding shock, and so on. Such irreversible processes are inevitably accompanied by entropy increase and energy dissipation. It is full of challenges and valuable to quantitatively analyze each of these phenomena.

In this section, we focused on the irreversible attenuations of the leading transmitted shock and of the reflected shock from the first perforated plate due to its important significance in practical applications. Generally, the passage of a shock wave is able to cause irreversible changes of local thermodynamic parameters at a location in the flow field. By using the Rankine–Hugoniot condition, an entropy increase that depends on the intensity of the shock wave can be expressed in a non-dimensional form as

(8) $$\left(\frac{\Delta s}{c_{v}}\right)=\ln\left(\frac{p_{A}}{p_{B}}\right)-\gamma \ln\left(\frac{1+\frac{\gamma +1}{\gamma -1}\frac{p_{A}}{p_{B}}}{\frac{\gamma +1}{\gamma -1}+\frac{p_{A}}{p_{B}}}\right)$$

where Δ*s* denotes the entropy increase; *c_v_* is the specific heat at constant volume; and *γ* represents the ratio of specific heats of air; and *p*_A_/*p*_B_ signifies the ratio of pressures after and before the passage of the shock wave. In cases of the incident, leading transmitted, and reflected shock waves, the pressure ratio should respectively be replaced with *p*_2_/*p*_1_, *p*_L_/*p*_1_ and *p*_R_/*p*_2_, here *p*_R_ denoting the post-shock pressure of the reflected shock wave.. The non-dimensional entropy increase is monotonously increased with increasing pressure ratio (for *p*_2_/*p*_1_ greater than unity) or intensity of the shock wave because the first derivative of entropy increase with regard to the pressure ratio is positive. In light of such a characteristic of the shock wave, in order to quantitatively analyze the effects of shock Mach number, porosity, and plate number on shock wave attenuation in another way, at this point, we would like to introduce an entropy-based attenuation rate as

(9) $$\beta_{s}=1-\frac{{\left(\Delta s/c_{v}\right)}_{E}}{{\left(\Delta s/c_{v}\right)}_{I}}$$

where (Δ*s*/*c_v_*)_I_ is the non-dimensional entropy increase caused by the incident shock wave and (Δ*s*/*c_v_*)_E_ is the exploratory non-dimensional entropy increase due to the leading transmitted or reflected shock wave, denoted by a subscript “L” or “R” respectively in the following analysis for clarity.

**Figure 13:**
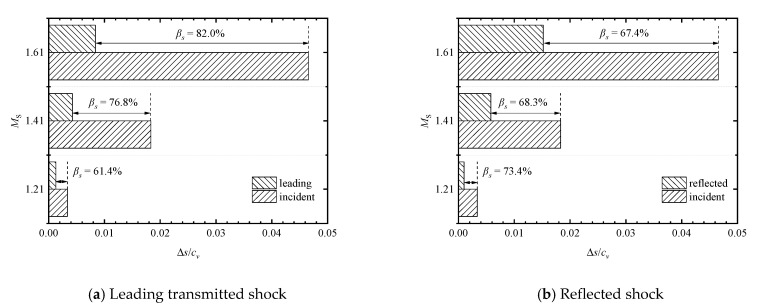
Comparisons of entropy-based attenuation rates of the leading transmitted and reflected shock waves at different incident shock Mach numbers.

Comparisons of pressure-based attenuation rates of the leading transmitted shock wave at different shock Mach numbers are shown in Figure 13a,b. In the *M*_s_ = 1.21 case, the non-dimensional entropy increases due to the incident, leading transmitted, and reflected shock waves are calculated as (Δ*s*/*c_v_*)_I_ = 3.35 × 10^−^^3^, (Δ*s*/*c_v_*)_L_ = 1.29 × 10^−^^3^, and (Δ*s*/*c_v_*)_R_ = 9.89 × 10^−^^4^, respectively, thus the entropy-based leading transmitted and reflected shock attenuation rates *β_s_ =* 61.4% and 73.4%, respectively. In the *M*_s_ = 1.41 case, the non-dimensional entropy increases are obtained as (Δ*s*/*c_v_*)_I_ = 1.83 × 10^−^^2^, (Δ*s*/*c_v_*)_L_ = 4.25 × 10^−^^3^, and (Δ*s*/*c_v_*)_R_ = 5.79 × 10^−^^3^, respectively, thus the shock attenuation rates *β_s_ =* 76.8% and 68.3%, respectively. In the *M*_s_ = 1.61 case, we have (Δ*s*/*c_v_*)_I_ = 4.66 × 10^−^^2^, (Δ*s*/*c_v_*)_L_ = 8.38 × 10^−^^3^, and (Δ*s*/*c_v_*)_R_ = 1.52 × 10^−^^2^, respectively, thus the shock attenuation rates *β_s_ =* 82.0% and 67.4%, respectively. This indicates that the increase in the incident shock Mach number increases the attenuation of the leading shock wave during its propagation through an array of perforated plates. This is consistent with the conclusion drawn from the preceding analysis of pressure-based attenuation rates, although the corresponding pressure- and entropy-based attenuation rates are quite different in magnitude because the entropy increase is not simply in direct proportion to pressure ratio. On the other hand, it can be concluded that the increase of the incident shock Mach number decreases the attenuation of the reflected shock wave from the first perforated plate of the array.

**Figure 14:**
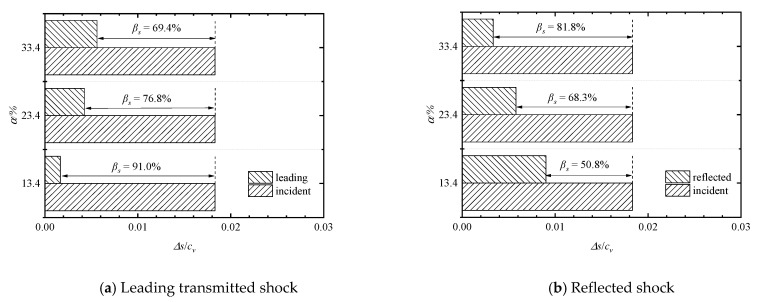
Comparisons of entropy-based attenuation rates of the leading transmitted and reflected shock waves at different porosities.

Comparisons of entropy-based attenuation rates of the leading transmitted and reflected shock waves for *M*_s_ = 1.41 at different porosities are shown in Figure 14a,b, respectively. It can be found that when *α* = 13.4%, 23.4%, and 33.4%, all the non-dimensional entropy increases due to the incident shock waves have the same value of 1.83 × 10^−^^2^, while the entropy increases due to the leading transmitted shock waves are 1.66 × 10^−^^3^, 4.25 × 10^−^^3^, and 5.61 × 10^−^^3^, and therefore the entropy-based shock attenuation rates are 91.0%, 76.8%, and 69.4%, respectively. Thus, we can make the same summary as above, that the decreasing porosity improves the inhibition effect of the perforated plates on the leading shock wave, and thus further decreases the shock intensity. On the other hand, the entropy increases due to the reflected shock waves are 9.01 × 10^−3^, 5.79 × 10^−3^, and 3.34 × 10^−3^, and therefore the entropy-based shock attenuation rates are 50.8%, 68.4%, and 81.8%, respectively. It indicates that the decrease of porosity decreases the attenuation of the reflected shock wave from the first perforated plate of the array.

**Figure 15:**
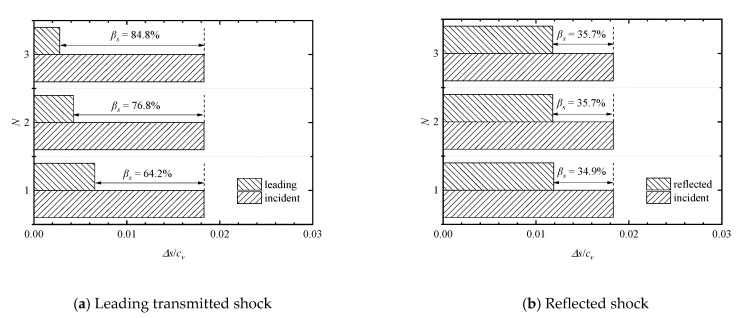
Comparisons of entropy-based attenuation rates of the leading transmitted and reflected shock waves at different plate numbers.

Comparisons of entropy-based attenuation rates of the leading transmitted and reflected shock waves for *M*_s_ = 1.41 at different plate numbers is shown in Figure 15a,b, respectively. It can be found that in the single-, double-, and triple-plate cases, the entropy increases due to the leading shock waves are 6.56 × 10^−^^3^, 4.25 × 10^−^^3^, and 2.78 × 10^−^^3^, and therefore, the entropy-based shock attenuation rates are 64.2%, 76.8%, and 84.8%, respectively. One can find that the entropy-based attenuation rate increases as the plate number increases. Furthermore, the entropy-based attenuation of the leading shock wave because of the subsequent addition of each plate was far less than that due to the first plate, meaning that the first plate has a crucial effect on the shock wave attenuation. This qualitative dependency is completely in accord with that obtained from the above analysis of attenuation rates based on pressures. On the other hand, the entropy increases due to the reflected shock waves are 1.19 × 10^−2^, 1.18 × 10^−2^, and 1.18 × 10^−2^, and therefore the entropy-based shock attenuation rates are 34.9%, 35.7%, and 35.7%, respectively. It means that the increase of plate number has negligible effect on the attenuation of the reflected shock wave from the first perforated plate of the array.

## 6. Conclusions

In this work, the propagation behavior of a planar incident shock wave through an array of perforated plates is numerically simulated. A parametric study was performed to analyze the dependence of parameters on the propagation and attenuation of the leading shock wave, and the related phenomena and mechanisms. The main conclusions are drawn as follows:

1. The intensity of the leading shock wave experiences a complex process consisting of attenuation, local enhancement, attenuation, enhancement, and attenuation because of the reflection, diffraction, transmission, and interference behaviors and the superimposed effects caused by the trailing second shock wave. The interactions of a reflected shock wave with transmitted shock-induced ring vortices and oblique shock patterns with jet flows cause the deformation and local intensification of the shock wave. An oscillation chamber for the inside dynamic wave system is created between two perforated plates, causing the formation of the following nearly steady jets. The vorticity diffusion, merging, and splitting of vortex cores contribute to the dissipation of wave energy.
2. The leading transmitted shock wave attenuates more significantly whereas the reflected shock wave from the first plate of the array attenuates less significantly as the incident shock Mach number increases. The decrease in perforated-plate porosity increases the attenuation rate of the leading shock wave due to the increasing suppression effects on the shock wave while decreases the attenuation rate of the reflected shock wave. The increase of plate number increases the attenuation rate of the leading shock wave but plays a negligible role in the attenuation rate of the reflected shock wave. The first perforated plate in the array has a major effect on the attenuation of the shock wave.

## Figures and Tables

**Table 1:** Parameters of perforated-plate models.

Type	*D*_h_/mm	*L*_h_/mm	*α*/%
A	1.89	4	13.4
B	2.50	4	23.4
C	2.98	4	33.4

**Table 2:** Parameters in the initial flow fields.

*M*_S_	*T*_2_/K	*p*_2_/MPa	*v*_2_/m·s^−1^	*T*_0_/K	*p*_0_/MPa
1.21	340.3	0.156	111.0	346.4	0.166
1.41	378.3	0.218	202.8	398.8	0.262
1.61	418.5	0.290	286.1	459.2	0.401

**Table 3:** Comparison of the peak values from the calculations on grid2 and from Ram’s experiment [26].

Peak No.	Computational *p*/*p*_1_	Experimental *p*/*p*_1_	Relative Error *E_r/_*%
1	2.48	2.40	3.33
2	2.79	2.78	0.36
3	3.48	3.40	2.35
4	3.71	3.72	0.27
5	4.15	4.17	0.24
